# The impact of young maternal age at birth on neonatal mortality: Evidence from 45 low and middle income countries

**DOI:** 10.1371/journal.pone.0195731

**Published:** 2018-05-23

**Authors:** Sarah Neal, Andrew Amos Channon, Jesman Chintsanya

**Affiliations:** 1 Department of Social Statistics and Demography, University of Southampton, Southampton, United Kingdom; 2 Chancellor College, University of Malawi, Zomba, Malawi; Universidad del Desarrollo, CHILE

## Abstract

**Objectives:**

This study explores the impact of early motherhood on neonatal mortality, and how this differs between countries and regions. It assesses whether the risk of neonatal mortality is greater for younger adolescent mothers compared with mothers in later adolescence, and explores if differences reflect confounding socio-economic and health care utilisation factors. It also examines how the risks differ for first or subsequent pregnancies.

**Methods:**

The analysis uses 64 Demographic and Health Surveys collected between 2005 and 2015 from 45 countries to explore the relationship between adolescent motherhood (disaggregated as <16 years, 16/17 years and 18/19 years) and neonatal mortality. Both unadjusted bivariate association and logistic regression are used. Regional level multivariate models that adjust for a range of socio-economic, demographic and health service utilisation variables are estimated. Further stratified models are created to examine the excess risk for first and subsequent births separately.

**Findings:**

The risk of neonatal mortality in all regions was markedly greater for infants with mothers under 16 years old, although there was marked heterogeneity in patterns between regions. Adjusting for socio-economic, demographic and health service utilisation variables did not markedly change the odds ratios associated with age. The increased risks associated with adolescent motherhood are lowest for first births.

**Conclusion:**

Our findings particularly highlight the importance of reducing adolescent births among the youngest age group as a strategy for addressing the problem of neonatal mortality, as well ensuring pregnant adolescents have access to quality maternal health services to protect the health of both themselves and their infants. The regional differences in increased risk are a novel finding which requires more exploration.

## Introduction

Each year, an estimated 16 million young women give birth between the ages of 15 and 19 years [1], and around a further million give birth before the age of 15 years [2]. Around 95% of these births are in low or middle income countries. Adolescent motherhood is associated with a range of adverse outcomes for both mother and infant [3–7] including increased neonatal mortality, which has been demonstrated in a range of settings in both developed and developing countries [8–15]. Globally neonatal deaths now make up 45% of all deaths in children under five years [16], and hence a focus on reducing mortality within this age group is essential. This is recognised in the indicator for the third Sustainable Development Goal [SDG] which states that all countries should reduce their neonatal mortality rate to at least as low as 12 per 1000 live births [17].

Relatively few studies disaggregate neonatal mortality by different adolescent age groups, leading to a gap in knowledge regarding the relationship between neonatal mortality and age throughout the adolescent period. Comparisons of results from studies that do differentiate by adolescent age are difficult due to the use of different cut-offs for age groups. Furthermore, most are focussed on either a single country or a small group of countries, which does not allow comparison across geographical regions. Several previous studies only identify risk at the lower end of the adolescent spectrum, such as for those under 16 years of age [3,12], with the neonates of older adolescents experiencing similar risks to those of mothers in their 20s. Comparison between regions will provide indication of a potential contextual element in the relationship between adolescent birth and neonatal mortality.

What is also unclear is whether poorer pregnancy outcomes in adolescence are a result of biological or physiological processes, or reflect other confounding socio-economic and health care utilisation factors. Women who give birth during adolescence are more likely to be less wealthy and have received less education [18] and, in some countries, may make less use of health services [19,20]. In addition it is more likely to be their first birth, which carries increased risks [21]. Sharma et al.’s study indicates that there is an inherent biological risk associated with young maternal age that is not mitigated by adjusting for socio-economic, biodemographic and health systems variables [12]. In comparison, other studies have concluded that there is no increased risk for adolescent mothers once adjustment has been made for such confounding factors [22].

This study provides a comprehensive, multi-country analysis of the association between adolescent age and neonatal mortality. We concentrate on how risk patterns differ for younger and older adolescents, examining the risk of neonatal death by individual year of mother’s age. We are interested in identifying a potential “tipping point” at the age where the risk of neonatal death decreases. This is particularly important as the regions of the world where very early adolescent motherhood is still prevalent (e.g. parts of sub-Saharan Africa and several Asian countries) are also those with high rates of neonatal mortality. Disaggregation of adolescent age groups provides a nuanced picture, indicating the possible benefits of focussing attention on reducing pregnancies in the youngest age groups, which has not received the emphasis it deserves [23].

Through the pooling of a large number of country datasets to produce regional estimates, some of the limitations in interpreting previous studies with smaller samples are overcome, whilst allowing for geographical comparison. We also undertake analysis adjusting for a range of possible confounding factors in order to indicate whether the association is driven by biological or physiological factors, or socio-economic, biodemographic and health service utilisation determinants. Further analysis examines whether birth in adolescence has a different impact on first or subsequent births.

## Methodology

The study is based on analysis of 64 Demographic and Household Surveys (DHS) from 45 countries carried out between 2005 and 2015 in low and middle income countries (see S1 Table for list of surveys). The DHS are large, nationally representative surveys that collect data on a range of demographic and health variables that are comparable across time and countries [24]. Thirty one countries were in sub-Saharan Africa, eight in South and South-East Asia and the remaining six were in Latin America and the Caribbean. In a number of countries more than one survey was available since 2005, and when this occurred, surveys were pooled to increase sample size. A cut-off of 2005 was included to ensure the datasets were reasonably contemporaneous while still providing a large sample of countries reflecting a wide geographic spread. Women aged 15 to 49 in sampled households provide a full birth history, with further questions about births that occurred within a defined period before the survey (usually of five years). This included the survival status of the child, with the age at death for those that died.

The outcome of analysis is neonatal mortality, defined as death within the first 28 days of life. Initial bivariate binary logistic regression was used to estimate unadjusted odds ratios (ORs) for neonatal mortality by age of the mother at the time of the birth. Age at birth was grouped as <16 years, 16/17 years and 18/19 years with a reference category of 20–29 years. Individual models were run for each country, as well as for data pooled at the regional and aggregate regional level in order to increase the sample size. The dependant variable for analysis of individual countries and small regions was neonatal mortality, but separate models for early and late mortality were developed for aggregated regions. Early neonatal mortality refers to a death of a live-born baby within the first seven days of life, while late neonatal mortality covers the time after 7 days until before 28 days. Further models were run for neonatal mortality with maternal age at birth in individual years (e.g. <15, 15, 16, 17, 18 and 19 years) for the aggregate regional level to examine patterns in more detail.

In addition, ORs were also calculated for models based on the aggregate regional level adjusted for a range of socio-economic, demographic and health service utilisation factors that are known to be associated with both adolescent motherhood and the risk of neonatal death. These include urban / rural residence, maternal education (grouped into no education, primary and secondary/tertiary), birth order (grouped into first birth, 2/3^rd^ birth and 4^th^ or higher birth), antenatal care utilisation (ANC; no ANC, 1–3 visits or 4 or more visits) and place of birth (home or facility). DHS surveys measure wealth through asset indices, which are a composite measure of a household's cumulative living standard. The index is calculated using data on a household’s ownership of selected assets, housing construction and access to water and sanitation facilities. However, these indices were not included in the analysis as they are not comparable across countries, although analysis using asset indices was included as part of the sensitivity analysis (not presented). Further separate stratified models were created for first births and subsequent births.

In order to account for the differential chances of households being selected into the surveys, sample weights were applied in all analyses. This was conducted within each country and survey. No weighting was conducted to reflect the population sizes of each country in the regional level estimates. Sensitivity testing was carried out by re-running the models with no weighting, and also using only one survey per country (as well as with asset index included) to check this was not introducing bias.

## Findings

### Initial descriptive statistics

Table 1 shows the number of births and neonatal deaths to women aged under 20 years grouped by region and age. In total the study included 4,116 deaths in the neonatal period to mothers aged under 20 years, 83% of which were in the early neonatal period. The table also shows the percentage of births for each age group that resulted in a neonatal death. In most regions there is a monotonic pattern with the percentage of births resulting in neonatal death increasing with decreasing maternal age, and this pattern is clear for sub-Saharan Africa and South and South-East Asia. However, in the Latin American and Caribbean region this pattern is somewhat less pronounced, with only a raised percentage of deaths in the <16 years maternal group.

**Table 1:** Number of births and neonatal deaths to mothers in each age group by region, and percentage of births ending in neonatal death (unweighted data).

	Births to mothers aged <16 years			Births to mothers aged 16/17 years			Births to mothers aged 18/19 years			Births to mothers aged 20/29 years		
	Neonatal deaths	All births	% births ending in neonatal death	Neonatal deaths	Total	% births ending in neonatal death	Neonatal deaths	All births	% births ending in neonatal death	Neonatal deaths	All births	% births ending in neonatal death
**Region**												
East Africa	95	1,821	5.2	301	7,088	4.2	459	12,681	3.6	1,887	72,949	2.6
West Africa	255	4,242	6.0	631	12,900	4.9	681	18,791	3.6	3,374	115,334	3.0
Middle Africa	29	943	3.1	87	2,823	3.1	125	4,315	2.9	533	20,950	2.5
South Africa	6	157	3.8	25	771	3.2	35	1,389	2.5	156	5,966	2.6
**sub-Saharan Africa**	**385**	**7163**	**5.4**	**1044**	**23582**	**4.4**	**1300**	**37176**	**3.5**	**5950**	**215199**	**2.8**
South Asia	94	1,556	6.0	307	5,370	5.7	521	11,051	4.7	1,905	62,037	3.1
South East Asia	11	151	7.3	40	1,079	3.7	85	2,893	2.9	438	23,143	1.9
**South and South East Asia**	**105**	**1707**	**6.2**	**347**	**6449**	**5.4**	**606**	**13944**	**4.3**	**2,343**	**85,180**	**2.8**
South America	28	1,181	2.4	40	3,409	1.2	66	4,896	1.3	264	21,478	1.2
Central America	12	573	2.1	33	1,733	1.9	44	2,499	1.8	142	10,815	1.3
Caribbean	11	522	2.1	44	1,675	2.6	51	2,461	2.1	273	12,427	2.2
**Latin America and the Caribbean**	**51**	**2276**	**2.2**	**117**	**6817**	**1.7**	**161**	**9856**	**1.6**	**679**	**44,720**	**1.5**
**Total all regions**	**541**	**11,146**	**4.85**	**1,508**	**36,848**	**4.09**	**2,067**	**60,976**	**3.39**	8,972	345,099	**2.6**

### Bivariate regression analysis

S2 Table contains unadjusted ORs for maternal age for each country with neonatal deaths as the outcome variable. While few of the estimates are statistically significant at country level (potentially due to the small numbers of deaths within each country) we see a very clear trend in most countries with markedly increased ORs for the <16 years grouping, with ORs then decreasing in size for the 16/17 and 18/19 age groups. In some cases the significantly increased OR for the <16 grouping is very large e.g. Indonesia, Ethiopia and Guyana, albeit with large confidence intervals (CIs). There are still significantly large increased odds of neonatal death in the 18/19 age group in some countries (e.g. Maldives, Malawi) compared to mothers aged 20–29. However, in the majority of countries the ORs for this age group are negligible or even reduced.

When we look at the pooled estimates by region (Table 2), we find clear and statistically significant patterns of high ORs for neonatal deaths for infants born to the youngest mothers which decrease with increasing maternal age for East Africa, West Africa, South Asia and South-East Asia. Southern Africa shows similar patterns, but the differences are not significant. In South and Central America and the Caribbean there is only evidence of increased ORs for the youngest group (and this is only significant for South and Central America).

**Table 2:** Unadjusted ORs for maternal age groups by region with neonatal mortality, early neonatal mortality and late neonatal mortality as outcome variable.

Total neonatal mortality	Mother's age at child birth (R.C.: 20–29)		
**Region**	**<16**	**16/17**	**18/19**
East Africa	1.90 (1.46–2.46)***	1.62 (1.38–1.89)***	1.36 (1.19–1.56)***
West Africa	2.06 (1.76–2.40)***	1.68 (1.52–1.86)***	1.25 (1.14–1.39)***
Middle Africa	1.15 (0.71–1.85)	1.06 (0.76–1.47)	1.35 (1.00–1.83)*
Southern Africa	1.37 (0.55–3.42)	1.23 (0.761.99)	1.01 (0.591.73)
South Asia	1.93 (1.49–2.49)***	1.73 (1.48–2.03)***	1.44 (1.27–1.64)***
Southeast Asia	5.54 (2.27–13.53)***	1.85 (1.24–2.77)***	1.60 (1.15–2.22)***
South / Central America	1.72 (1.13–2.61)*	1.12 (0.83–1.53)	1.04 (0.80–1.35)
Caribbean	1.43 (0.65–3.15)	1.14 (0.71–1.82)	0.79 (0.52–1.19)
Sub-Saharan Africa	1.91 (1.69–2.17)***	1.58 (1.46–1.72)***	1.29 (1.19–1.39)***
South &Southeast Asia	2.26 (1.77–2.89)***	1.86 (1.61–2.16)***	1.52 (1.35–1.71)***
Latin American & Caribbean	1.57 (1.08–2.29)*	1.10 (0.85–1.43)	0.93 (0.75–1.16)
**Early neonatal mortality**	**<16**	**16/17**	**18/19**
East Africa	2.11 (1.60–2.78)***	1.71 (1.45–2.03)***	1.44 (1.25–1.67)***
West Africa	2.03 (1.72–2.39)***	1.67 (1.50–1.87)***	1.18 (1.05–1.32)***
Middle Africa	1.02 (0.60–1.74)	0.92 (0.66–1.29)	1.39 (1.00–1.92)*
Southern Africa	1.57 (0.63–3.96)	1.22 (0.72–2.05)	1.07 (0.60–1.90)
South Asia	1.98 (1.50–2.62)***	1.76 (1.48–2.09)***	1.49 (1.30–1.71)***
Southeast Asia	6.03 (2.41–15.09)***	1.68 (1.07–2.65)*	1.48 (1.05–2.10)*
South / Central America	1.70 (1.07–2.70)*	0.99 (0.70–1.40)	0.96 (0.72–128)
Caribbean	1.92 (0.87–4.25)	0.87 (0.52–1.46)	0.84 (0.52–1.34)
Sub-Saharan Africa	1.93 (1.69–2.21)***	1.59 (1.46–1.74)***	1.28 (1.18–1.40)***
South & Southeast Asia	2.32 (1.77–3.02)***	1.85 (1.57–2.18)***	1.54 (1.36–1.75)***
Latin American & Caribbean	1.68 (1.12–2.51)*	0.95 (0.72–1.26)	0.94 (0.74–1.19)
**Late neonatal mortality**	**<16**	**16/17**	**18/19**
East Africa	1.0 (0.46–2.18)	1.15 (.77–1.72)	0.92 (0.65–1.31)
West Africa	2.16 (1.52–3.10)***	1.68 (1.31–2.17)***	1.61 (1.29–2.01)***
Middle Africa	1.94 (0.87–4.33)	1.91 (0.84–4.36)	1.11 (0.61–2.03)
Southern Africa	omitted	1.31 (0.43–4.01)	0.77 (0.24–2.47)
South Asia	1.59 (0.87–2.91)	1.55 (1.12–2.16)**	1.23 (0.94–1.62)
Southeast Asia	1.55 (0.21–11.41)	3.01 (1.30–6.97)*	2.38 (.98–5.71)
South / Central America	1.67 (0.63–4.3)	1.63 (0.87–3.07)	1.35 (0.79–2.30)
Caribbean	omitted	1.94 (0.81–4.65)	0.64 (0.29–1.45)
Sub-Saharan Africa	1.81 (1.34–2.43)***	1.52 (1.24–1.88)***	1.29 (1.08–1.54)**
South &Southeast America	1.87 (1.04–3.38)*	1.80 (1.07–1.81)***	1.39 (1.07–1.81)*
Latin American & Caribbean	0.92 (0.35–2.38)	1.71 (0.99–2.91)	0.63–1.57)

Significance level:

*p≤0.05;

**p≤0.01;

***p≤0.005

Comparable findings are found when the analysis is run with early neonatal deaths as the outcome. For late neonatal deaths only West Africa has a significantly increased OR for all age groups, thought to be due to small sample sizes. When regions are further amalgamated into just three large regions patterns remain clear and significant for sub-Saharan Africa and South and South East Asia. While there is a gradient, there are statistically increased odds for neonatal deaths within all adolescent age groups compared with women aged 20–29 years. However in Latin America and the Caribbean, there is only a raised OR of neonatal deaths for mothers under 16 years. Models using early and late neonatal deaths as the outcome variables showed very similar ORs for sub-Saharan Africa and South and South-East Asia, but no ORs were significant in Latin America and the Caribbean.

When examining ORs by mother’s age in single years (Table 3) we found a very clear monotonic pattern for sub-Saharan Africa. In South and South East Asia there was a pattern of significantly increased risk that decreased by age from ages 15–19 years, but for the <15 years group the OR was not significant. In Latin America the ORs were only markedly increased for those aged <15 years and 15 years, but were only significant for those aged 15 years. The total number of deaths by age and large regional grouping are shown in S3 Table.

**Table 3:** Unadjusted O.R. for neonatal deaths by mother's age at birth of child in individual years and by regions.

	Neonatal deaths		
Mother's age at child birth	Sub-Saharan Africa	Southeast Asia	Latin America & Caribbean
<15	2.28 (1.87–2.79)***	1.61 (0.99–2.60)	1.54 (0.79–2.99)
15	1.73 (1.48–2.02)***	2.53 (1.91–3.35)***	1.59 (1.01–2.48)*
16	1.71 (1.52–1.92)***	1.87 (1.50–2.35)***	1.08 (0.75–1.57)
17	1.50 (1.35–1.67)***	1.86 (1.55–2.22)***	1.12 (0.80–1.57)
18	1.34 (1.20–1.50)***	1.54 (1.31–1.81)***	0.89 (0.65–1.21)
19	1.24 (1.13–1.38)***	1.51 (1.30–1.75)***	0.97 (0.73–1.29)
20–29	1.00 (Reference)	1.00 (Reference)	1.00 (Reference)

Significance level:

*p≤0.05;

**p≤0.01;

***p≤0.005

### Multivariate regression analysis

The multivariate analysis explores whether the relationship between age and neonatal death is attenuated by the addition to the model of other indicators known to be related to mortality. The percentage distribution of these indicators, by large regional grouping, is shown in S4 Table. Three further models were created for each large region grouping (Tables 4–6): in model 2 we added birth order and birth interval, in model 3 we added socio-economic variables (education and place of residence) and in model 4 we added health service utilisation variables (place of delivery and ANC). In SSA the point estimate for the OR is attenuated slightly for the <16 age group for models 2–4, whereas ORs are unchanged for the other age groups. For Asia the ORs are attenuated slightly for all age groups, with the largest effect between models 2 and 3 (when education is added). In Latin America only maternal age at birth of <16 years was significant, and the addition of other possible confounding variables only produced a modest reduction in OR.

**Table 4:** Adjusted O.R. for neonatal deaths by mother's age at birth of child: Sub-Saharan Africa.

	Model 2	Model 3	Model 4
**Maternal age (R.C. = 20–29)**			
**< 16 years**	1.63	1.54	1.48
	(1.42–1.86)**	(1.34–1.76)**	(1.29–1.70)**
**16/17 years**	1.38	1.32	1.32
	(1.26–1.52)**	(1.21–1.45)**	(1.20–1.46)**
**18/ 19 years**	1.18	1.15	1.19
	(1.08–1.29)**	(1.06–1.26)**	(1.09–1.30)**
**Birth order (R.C. 2/3)**			
**First birth**	1.52	1.59	1.49
	(1.41–1.63)**	(1.48–1.71)**	(1.39–1.60)**
**4+ birth**	1.28	1.23	1.25
	(1.18–1.40)**	(1.13–1.34)**	(1.14–1.36)**
**Birth interval (R.C. >18 months)**			
**Birth interval <18months**	1.83	1.83	1.62
	(1.70–1.96)**	(1.70–1.96)**	(1.50–1.74)**
**Education (R.C. secondary / tertiary education)**			
**No education**		1.26	1.12
		(1.16–1.37)**	(1.03–1.23)*
**Primary education**		1.20	1.13
		(1.10–1.31)**	(1.03–1.27)**
**Place of residence (R.C. urban)**			
**Rural**		1.05	1.01
		(0.98–1.13)	(0.94–1.09)
**Place of delivery (R.C. home)**			
**Hospital**			1.00
			(0.94–1.07)
**Antenatal care (R.C. 4 or more)**			
**No ANC**			1.39
			(1.23–1.56)**
**<4 ANC**			1.13
			(1.02–1.24)*
**Data missing**			2.82
			(2.62–3.03)**

Significance level:

*p<0.05;

**p<0.01;

***p<0.005

**Table 5:** Adjusted O.R. for neonatal deaths by mother's age at birth of child: South and South East Asia.

	Model 2	Model 3	Model 4
**Maternal age (R.C. = 20–29)**			
**< 16 years**	2.12	1.84	1.81
	(1.65–2.74)**	(1.43–2.38)**	(1.37–2.37)**
**16/17 years**	1.75	1.55	1.49
	(1.50–2.04)**	(1.33–1.81)**	(1.26–1.76)**
**18/ 19 years**	1.46	1.35	1.30
	(1.29–1.65)**	(1.20–1.53)**	(1.14–1.48)**
**Birth order (R.C. 2/3)**			
**First birth**	1.56	1.802	1.532
	(1.400–1.740)**	(1.611–2.016)**	(1.353–1.734)**
**4+ birth**	1.76	1.47	1.46
	(1.50–2.06)**	(1.27–1.74)**	(1.24–1.73)**
**Birth interval (R.C. >18 months)**			
**Birth interval <18 months**	2.24	2.23	1.92
	(1.99–2.52)**	(1.98–2.51)**	(1.70–2.18)**
**Education (R.C. secondary/ tertiary education)**			
**No education**		1.77	1.45
		(1.59–1.98)**	(1.28–1.64)**
**Primary education**		1.35	1.24
		(1.19–1.53)**	(1.09–1.41)**
**Place of residence (R.C. urban)**			
**Rural**		1.26	1.28
		(1.13–1.41)**	(1.14–1.44)**
**Place of delivery (R.C. home)**			
**Hospital**			1.28
			(1.14–1.43)**
**Antenatal care (R.C. 4 or more)**			
**No ANC**			1.58
			(1.30–1.92)**
**<4 ANC**			1.44
			(1.22–1.69)**
**Data missing**			3.91
			(3.40–4.50)**

Significance level:

*p<0.05;

**p<0.01;

***p<0.005

**Table 6:** Adjusted O.R. for neonatal deaths by mother's age at birth of child: Latin America and the Caribbean.

	Model 2	Model 3	Model 4
**Maternal age (R.C. = 20–29)**			
**< 16 years**	1.69	1.64	1.56
	(1.14–2.50)**	(1.10–2.45)*	(1.01–2.41)*
**16/17 years**	1.15	1.13	1.14
	(0.88–1.50)	(0.86–1.49)	(0.85–1.54)
**18/ 19 years**	0.95	0.95	0.91
	(0.76–1.20)	(0.75–1.19)	(0.71–1.16)
**Birth order (R.C. 2/3)**			
**First birth**	1.05	1.08	1.16
	(0.86–1.28)	(0.88–1.34)	(0.93–1.44)
**4+ birth**	1.41	1.31	1.18
	(1.01–1.95)*	(0.93–1.83)	(0.82–1.71)
**Birth interval (R.C. >18 months)**			
**Birth interval <18 months**	1.66	1.65	1.48
	(1.32–2.07)**	(1.32–2.07)**	(1.16–1.89)**
**Education (R.C. secondary/tertiary education)**			
**No education**		1.46	0.96
		(1.06–2.01)*	(0.66–1.38)
**Primary education**		1.04	0.89
		(0.85–1.28)	(0.71–1.12)
**Place of residence (R.C. urban)**			
**Rural**		1.07	0.88
		(0.89–1.28)	(0.72–1.07)
**Place of delivery (R.C. home)**			
**Hospital**			0.72
			(0.57–0.89)**
**Antenatal care (R.C. 4 or more)**			
**No ANC**			2.62
			(1.81–3.80)**
**<4 ANC**			2.21
			(1.67–2.93)**
**Data missing**			3.33
			(2.76–4.08)**

Significance level:

*p<0.05;

**p<0.01;

***p<0.005

The sensitivity analyses, estimating the results with no weighting or only using one survey per country, showed no large change in ORs (although in some cases there were some modest changes in the ORs and CIs). While asset wealth was not included as it is not comparable across countries, its addition did not result in any real change to the ORs for age at birth for any of the models.

### Stratified models for first and subsequent births

Table 7 shows the unadjusted and adjusted ORs for age at first birth for models carried out separately on first and subsequent births. For sub-Saharan Africa there were significantly increased ORs for first and subsequent births for age groups <16 years and 16/17 years for the unadjusted models (although confidence intervals are wide), but there was little difference in the adjusted models. The OR for first births was not increased for the 18/19 year age group for first births, but there was a significant increase in odds of neonatal death for subsequent births, albeit with a smaller increase than for younger ages. For South and South East Asia the OR were markedly larger for <16 and 16/17 year age groups for subsequent births in both the adjusted and unadjusted models, but again confidence intervals are relatively wide and overlap. ORs were similar for 18/19 age group, and were actually lower for subsequent births in the unadjusted model. In Latin America and the Caribbean there was only evidence of increased ORs for either first or subsequent births in the <16 group, but ORs were markedly larger for subsequent births, although again the confidence interval is wide.

**Table 7:** Unadjusted and adjusted O.R. for neonatal deaths by mother's age at birth of child disaggregated by first and subsequent births: All regions (adjusted model includes education, place of residence, place of delivery and ANC). Reference category = 20–29 years.

	First birth		Subsequent births	
	Unadjusted	Adjusted	Unadjusted	Adjusted
**Sub-Saharan Africa**				
**< 16 years**	1.52	1.33	2.07	1.39
	(1.32–1.75)**	(1.14–1.54)**	(1.35–3.18)**	(0.87–2.23)
**16/17 years**	1.23	1.15	1.82	1.49
	(1.11–1.37)**	(1.03–1.29)*	(1.54–2.14)**	(1.26–1.77)**
**18/ 19 years**	1.07	1.07	1.30	1.21
	(0.95–1.19)	(0.95–1.20)	(1.15–1.46)**	(1.07–1.36)**
**South / South East Asia**				
**< 16 years**	2.04	1.51	3.85	3.66
	(1.56–2.66)**	(1.14–2.01)**	(1.87–7.94)**	(1.70–7.88)**
**16/17 years**	1.68	1.28	2.33	2.08
	(1.40–2.00)**	(1.05–1.55)*	(1.76–3.07)**	(1.55–2.79)**
**18/ 19 years**	1.52	1.26	1.41	1.25
	(1.31–1.76)**	(1.07–1.48)**	(1.15–1.72)**	(1.01–1.54)*
**Latin America and Caribbean**				
**< 16 years**	1.60	1.43	3.70	3.26
	(1.06–2.43)*	(0.88–2.30)	(1.44–9.53)**	(1.10–9.71)*
**16/17 years**	1.20	1.18	1.19	0.90
	(0.87–1.65)	(0.81–1.71)	(0.71–2.00)	(0.51–1.56)
**18/ 19 years**	1.00	0.94	0.95	0.82
	(0.74–1.34)	(0.67–1.30)	(0.67–1.35)	(0.57–1.18)

Significance level:

*p<0.05;

**p<0.01;

***p<0.005

## Discussion

The results indicate that there are higher risks of mortality to neonates born to adolescent mothers, both before and after adjusting for confounding factors. This suggests that adolescent motherhood is associated with a potential physiological risk of neonatal mortality, which is particularly concentrated among younger teens. In some cases the increased risk can be marked: the risk of neonatal mortality in South and South-East Asia is over 100% greater for children of mothers under the age of 16 compared with those aged 20–29, even after adjusting for potential confounders.

A number of possible physiological pathways for how young maternal age impacts on neonatal mortality have been established or hypothesised. There is clear evidence that adolescent mothers are more likely to have babies that are low birth weight or born prematurely [10], both of which are important causes or contributing factors to newborn mortality [25]. Studies have differed in the magnitude of the increased mortality risk for children born to adolescent mothers due to the age groups and methodologies used. This increased risk may partly be underpinned by nutritional insufficiencies: there is a theory that adolescent pregnancies may involve mother-foetus competition for nutrients as younger adolescents in particular may still be requiring additional energy for growth [26]. There is also evidence that adolescents may be at greater risk of certain infections including malaria [27] and urinary tract infections [28], which can also affect birth weight and length of gestation. A number of studies have also found increased risk of hypertension in pregnancy in adolescents [4,29] and a further study found this increase to be most pronounced for those under the age of 16 years [3]: this important cause of maternal morbidity is also associated with poor neonatal outcomes [30]. Further studies suggest risk associated with physiological immaturity: reduced blood supply to the uterus and cervix may make infection more common [9], and there is also evidence that adolescents have reduced placental transportation [31]. This study cannot identify the underlying reason for the increased neonatal mortality to young adolescent mothers, nor whether the physiological pathways are the same in each of the regions.

An important aspect of this paper is the ability to compare findings between regions in order to assess if the factors underlying the risks associated with adolescent motherhood vary. The geographical variation observed is one of the most striking findings from this study. While in sub-Saharan Africa and South and South-East Asia there is evidence of risk throughout the adolescent period (albeit reducing with age] in Latin America increased risk appears to be only found in the <16 years group, which corresponds with Conde-Agudelo’s findings [8]. This could reflect regional differences in risk factors such as nutritional deficiency or causes of maternal morbidity. Firstly in some cases the incidence of maternal health conditions underlying the increased risk of mortality may vary by region: Malaria transmission rates are much higher in large parts of sub-Saharan Africa and South Asia compared to Latin America and the Caribbean where transmission rates are relatively low. Secondly, the nutritional status of adolescent girls varies markedly between regions: for instance the proportion of women of reproductive age who suffer from anaemia (a condition associated with increased risk of low birth weight and perinatal death) [32] is higher in much of sub Saharan Africa and Asia in comparison to Latin America and the Caribbean [33] which may provide at least a partial explanation for these differential outcomes.

Underlying causes of disparity could be compounded by the fact that studies suggest increased risk to mother and baby is more strongly linked to gynaecologic (i.e. the time since menarche) rather than chronological age [34], which may vary across regions. While there is no comparable international data on age at menarche, decline in age of menarche is strongly negatively associated with female life expectancy [35]: female life expectancy is markedly higher for Latin America than the other two regions [36], so it would be expected that on average the gynaecologic age at time of birth of the Latin America women is relatively greater than those in the other regions. Earlier onset of menarche is likely to reduce maternal foetal competition for nutrients. However, further studies are needed to examine the underlying causes of these regional differences.

In all three regions the risk associated with adolescent motherhood was, in general, greater for second or subsequent adolescent births in comparison with first adolescent births. Similar findings have been documented in higher income countries [37–39] and can be explained by a high rate of low birth weight and prematurity. Adolescents who have subsequent births before aged 20 years are likely to be among the most deprived: while our study adjusts for some elements of socio-economic status it is not able to fully acknowledge all aspects of disadvantage.

### Limitations

While DHS surveys provide some of the highest quality standardised data in low and middle income settings, there are still limitations as the data gathered is retrospective. There is likely to be under-reporting of neonatal deaths [40], and very early neonatal deaths may be incorrectly categorised as stillbirths [41]. “Heaping” of deaths at age seven days may make differentiation between early and late deaths difficult [42]. Age shifting of respondents is also common which can have subsequent implications for the accuracy of age at birth [43]. There are suggestions that, particularly in sub-Saharan Africa, younger adolescent mothers may overstate their age at the time of survey [43]. If this is the case the pattern for sub-Saharan Africa may be more marked, with somewhat lower risks for the older groups once those with overstated age have been removed. It is also impossible to discount that some of the regional differences could reflect differential issues in reporting of very young births.

A further limitation is that neonatal death is only one outcome that could be investigated. Studying miscarriage and stillbirth would have given a more comprehensive picture of possible risks to adolescents. However, while some DHS do report stillbirths, the quality of the data is known to be poor, with significant under-reporting [44].

Finally, ideally our study would also adjust for a wider range of factors which may reflect social and environmental disadvantage. These would include smoking and alcohol consumption (where relevant), nutrition during pregnancy, nutritional status at time of pregnancy, and more complex measure of deprivation including a measure of social capital. Most DHS measure women’s body mass index (BMI), although measured at the time of the survey. As BMI is likely to have changed between the time of the pregnancy and the survey we did not include this as a control. In addition, while we adjust for use of health services, we cannot include a marker for the quality of care received, which may also be relevant. Such detailed information could only be gathered from longitudinal studies, but would provide more detailed information of the pathways through which disadvantage occurs.

### Policy relevance and recommendations

Our study clearly demonstrates the important link between the adolescent sexual health and child health agendas. According to these results, the prevention of adolescent pregnancies will have marked impact on the reduction of neonatal deaths, and accelerate progress towards the neonatal SDG target, with the greatest gain amongst reducing births in the most vulnerable under 16 age group. We strongly suggest that these findings emphasize the need for efforts to be focussed on reducing births in very young adolescents in countries where this is prevalent. Thirteen percent of women in West Africa and 9% of women in East Africa have given birth before the age of 16 years and in some countries the rates are particularly high: for instance, in Guinea, Mali and Niger 20% or more of women still give birth before the age of 16 years [45]. Little progress has been made in reducing this figure over the last few decades. Sub-Saharan Africa has a high rate of neonatal mortality (29 per 1000 live births [46]), so there are clear advantages for child health outcomes by addressing very early adolescent motherhood.

Reducing adolescent births will require co-ordinated efforts from the social development and education sectors, in addition to the development of comprehensive adolescent reproductive health services. In many circumstances it will also require links with efforts to eliminate child marriage, and, particularly in the case of very early adolescent births, improve child protection. Very young adolescent are often at a very different stage of cognitive development compared to their older peers, which means different approaches to improving sexual health outcomes are needed that are specifically designed to meet the needs of this group [47]. Young women who become pregnant in early adolescence are also more likely to be poor and not in school [48], which presents further challenges in ensuring those most at risk of very early pregnancy are reached with comprehensive sexual health education and services. It is also vital that adolescents have access to adequate maternal health care that recognises the increased risks that their infants face, including at second birth, which may not be fully recognised. There is ample evidence that pregnant adolescents in many contexts face stigma and discrimination when seeking care (49], and the development of sensitive and respectful services that meet their needs is essential in ensuring this.

In addition, this study points to the importance of access to maternal health care for adolescents during pregnancy and delivery. Many of the possible pathways through which increased neonatal mortality is transmitted can be managed or ameliorated through high quality and appropriate care: antenatal care packages which include nutritional interventions, intermittent presumptive treatment of malaria and detection and prompt treatment for eclampsia would address some of the potential underlying factors that may be causing the increased risk. While a recent study in West Africa demonstrated that most adolescent pregnant with their first infant received some ANC, the findings suggested that they seek care later, make fewer visits during pregnancy, and receive fewer components of care than older first-time mothers. This suggests efforts need to be made to reduce barriers and increase quality of care for this vulnerable group.

## Conclusion

This large scale analysis indicates there is an increase in the risk of neonatal deaths to adolescent mothers, with the risk most marked in the youngest age group. There is geographical variation in these risks, which may reflect differences in age at menarche, nutritional status or risk factors for maternal morbidities that underpin neonatal mortality. The increased risk associated with adolescent births was greater for second or subsequent births. These risks remained even after adjusting for socio-economic, demographic and health service utilisation factors.

Our findings highlight the importance of reducing adolescent births as a strategy for addressing the problem of neonatal mortality in order to make firm progress towards the neonatal SDG goal.

## Supporting information

S1 Table Surveys included in the study, with numbers of birth by age of mother <20 years. (DOCX)

S2 Table Unadjusted ORs for maternal age for each country with neonatal deaths as the outcome variable (R.C. = 20–29 years). (DOCX)

S3 Table Number of deaths and births by large regional grouping (unweighted). (DOCX)

S4 Table Percentage distribution of background factors by large regional grouping (weighted). (DOCX)
