# Supplementary material for: The impact of young maternal age at birth on neonatal mortality: Evidence from 45 low and middle income countries
Source: PLoS One. 2018 May 23;13(5):e0195731. doi: 10.1371/journal.pone.0195731 (PMC5965834; doi:10.1371/journal.pone.0195731)
Supplement: S1 Table — (DOCX) [file pone.0195731.s001.docx]

**Supplementary 1 Table**: Surveys included in the study, with numbers of birth by age of mother <20 years

|  | | | | | |
| --- | --- | --- | --- | --- | --- |
| Country | Year of survey | Age of mother at birth of child | | | |
|  |  | <16 | 16/17 | 18/19 | Total |
|  |  |  |  |  |  |
| Bangladesh | 2007 | 382 | 690 | 925 | 1997 |
|  | 2011 | 412 | 984 | 1389 | 2785 |
| Benin | 2006 | 168 | 589 | 1120 | 1876 |
|  | 2011-12 | 112 | 465 | 879 | 1456 |
| Bolivia | 2008 | 146 | 513 | 770 | 1429 |
| Burkina Faso | 2010 | 100 | 681 | 1470 | 2251 |
| Burundi | 2010 | 25 | 147 | 529 | 701 |
| Cambodia | 2005 | 20 | 204 | 601 | 825 |
|  | 2010 | 14 | 195 | 595 | 804 |
| Cameroon | 2011 | 282 | 811 | 1189 | 2283 |
| Colombia | 2005 | 377 | 1099 | 1590 | 3066 |
|  | 2010 | 466 | 1329 | 1907 | 3702 |
| Comoros | 2012 | 45 | 144 | 238 | 428 |
| Congo (Brazzaville) | 2005 | 84 | 341 | 575 | 1001 |
|  | 2011-12 | 189 | 566 | 819 | 1574 |
| Congo Democratic Republic | 2007 | 131 | 407 | 904 | 1442 |
| Cote d'Ivoire | 2011-12 | 169 | 481 | 645 | 1295 |
| Dominican Republic | 2007 | 317 | 893 | 1237 | 2447 |
| Ethiopia | 2005 | 180 | 619 | 910 | 1709 |
|  | 2011 | 118 | 535 | 871 | 1524 |
| Gabon | 2012 | 138 | 362 | 530 | 1030 |
| Ghana | 2008 | 27 | 101 | 204 | 332 |
| Guinea | 2005 | 184 | 427 | 518 | 1129 |
|  | 2012 | 266 | 587 | 700 | 1553 |
| Guyana | 2009 | 60 | 142 | 254 | 456 |
| Haiti | 2005-06 | 75 | 294 | 454 | 822 |
|  | 2012 | 85 | 349 | 594 | 1028 |
| Honduras | 2005-6 | 232 | 784 | 1196 | 2212 |
|  | 2011-12 | 318 | 830 | 1167 | 2315 |
| India | 2005-06 | 889 | 3451 | 7461 | 11800 |
| Indonesia | 2012 | 54 | 365 | 1093 | 1512 |
| Kenya | 2008-09 | 70 | 301 | 577 | 949 |
| Lesotho | 2009 | 37 | 249 | 494 | 780 |
| Liberia | 2007 | 101 | 325 | 526 | 951 |
|  | 2013 | 157 | 493 | 700 | 1349 |
| Madagascar | 2008-09 | 412 | 978 | 1266 | 2656 |
| Malawi | 2010 | 215 | 1118 | 2224 | 3557 |
| Maldives | 2009 | 2 | 17 | 144 | 163 |
| Mali | 2006 | 381 | 1042 | 1448 | 2872 |
|  | 2012-13 | 312 | 684 | 908 | 1904 |
| Mozambique | 2011 | 266 | 846 | 1174 | 2287 |
| Namibia | 2006-07 | 65 | 251 | 471 | 787 |
| Nepal | 2006 | 51 | 354 | 741 | 1146 |
|  | 2011 | 43 | 345 | 702 | 1090 |
| Niger | 2006 | 244 | 734 | 840 | 1819 |
|  | 2012 | 232 | 849 | 1142 | 2223 |
| Nigeria | 2008 | 510 | 1527 | 2096 | 4134 |
|  | 2013 | 510 | 1730 | 2469 | 4709 |
| Pakistan | 2006-07 | 53 | 267 | 631 | 952 |
|  | 2012 | 42 | 264 | 773 | 1079 |
| Rwanda | 2005 | 10 | 110 | 408 | 528 |
|  | 2010 | 14 | 103 | 438 | 555 |
| Sao Tome and Principe | 2008-09 | 18 | 106 | 160 | 284 |
| Senegal | 2005 | 188 | 587 | 878 | 1653 |
|  | 2010-11 | 217 | 582 | 919 | 1718 |
| Sierra Leone | 2008 | 135 | 326 | 509 | 970 |
|  | 2013 | 305 | 798 | 1162 | 2265 |
| Swaziland | 2006 | 48 | 248 | 360 | 657 |
| Tanzania | 2010 | 79 | 419 | 779 | 1276 |
| Timor-Leste | 2009-10 | 24 | 177 | 536 | 737 |
| Uganda | 2006 | 86 | 432 | 908 | 1426 |
|  | 2011 | 102 | 426 | 807 | 1334 |
| Zambia | 2007 | 73 | 352 | 667 | 1092 |
| Zimbabwe | 2005-06 | 53 | 314 | 699 | 1065 |
|  | 2010 | 62 | 343 | 673 | 1078 |
| **Total** |  | **11180** | **37083** | **62564** | **110827** |
