# Supplementary material for: The impact of young maternal age at birth on neonatal mortality: Evidence from 45 low and middle income countries
Source: PLoS One. 2018 May 23;13(5):e0195731. doi: 10.1371/journal.pone.0195731 (PMC5965834; doi:10.1371/journal.pone.0195731)
Supplement: S2 Table — (DOCX) [file pone.0195731.s002.docx]

**Supplementary 2 Table.** Unadjusted ORs for maternal age for each country with neonatal deaths as the outcome variable (R.C. = 20-29 years).

|  |  | **Neonatal mortality** |  |  |
| --- | --- | --- | --- | --- |
|  | <16 | 16/17 | 18/19 |  |
| **Region** | O.R. (C.I) | O.R. (C.I) | O.R. (C.I) |  |
| **East Africa** |  |  |  |  |
| Burundi | 4.19 (1.03-16.98)* | 1.24 (0.5-3.11) | 1.18 (0.68 -2.05) |  |
| Comoros | 1.50 (0.27-8.21) | 1.49 (0.48-4.57) | 1.93 (0.73-5.11) |  |
| Ethiopia | 2.03 (1.01-4.11)* | 1.88 (1.31-2.7)*** | 1.77 (1.28-2.45)*** |  |
| Kenya | 0.60 (0.14-2.47) | 1.70 (0.71-4.08) | 1.02 (0.41-2.55) |  |
| Madagascar | 2.20 (1.19-4.06)** | 1.14 (0.63-2.05) | 1.32 (0.86-2.02) |  |
| Malawi | 2.34 (1.32-4.16)*** | 1.49 (0.97-2.29) | 1.77 (1.26-2.48)*** |  |
| Mozambique | 1.51 (0.80-2.85) | 1.49 (0.96-2.33) | 1.26 (0.83-1.9) |  |
| Rwanda | 3.60 (0.83-15.55) | 1.95 (0.96-4.00) | 1.41 (0.95-2.1) |  |
| Tanzania | 2.16 (0.59-7.96) | 1.64 (0.85-3.18) | 1.22 (0.70-2.12) |  |
| Uganda | 2.60 (1.27-5.31)** | 1.81 (1.14-2.86)** | 1.37 (0.94-2.00) |  |
| Zambia | 1.63 (0.59-4.46) | 2.69 (1.60-4.51)*** | 0.76 (0.41-1.41) |  |
| Zimbabwe | 0.95 (0.22-4.07) | 1.39 (0.82-2.37) | 0.97 (0.62-1.53) |  |
| **West Africa** |  |  |  |  |
| Benin | 2.51 (1.44 -4.38)*** | 1.76 (1.25-2.49)*** | 1.23 (0.92-1.65) |  |
| Burkina Faso | 2.62 (1.17-5.86)* | 2.58 (1.69-3.93)*** | 1.55 (1.07-2.24)* |  |
| Cote d'Ivoire | 1.41(0.46-4.32) | 1.13 (0.62-2.06) | 1.28 (0.80-2.06) |  |
| Ghana | 3.65 (0.79-16.92) | 0.90 (0.26-3.07) | 0.96 (0.35-2.62) |  |
| Guinea | 1.23 (0.74-2.04) | 1.68 (1.19-2.37)** | 1.00 (0.65-1.56) |  |
| Liberia | 1.68 (0.89-3.20) | 1.11 (0.69-1.78) | 1.03 (0.66-1.61) |  |
| Mali | 2.14 (1.48-3.1)*** | 1.66 (1.25-2.2)*** | 1.17 (0.88-1.55) |  |
| Niger | 2.27 (1.36-3.79)*** | 1.58 (1.11-2.25)** | 1.74 (1.19-2.55)*** |  |
| Nigeria | 1.78 (1.33-2.38)*** | 1.87 (1.54-2.27)*** | 1.18 (1.00-1.41) |  |
| Senegal | 2.13 (1.31-3.46)*** | 1.44 (1.04-2.00)* | 1.32 (0.94-1.84) |  |
| Sierra Leone | 2.64 (1.65-4.24)*** | 1.4 (1.01-1.95)* | 1.25 (0.87-1.81) |  |
| **Middle Africa** |  |  |  |  |
| Cameroon | 1.62 (0.72-3.66) | 1.17 (0.71-1.94) | 1.35 (0.87-2.11) |  |
| Congo (Brazzaville) | 1.09 (0.49-2.42) | 0.71 (0.33-1.51) | 1.44 (0.82-2.53) |  |
| Congo Democratic Republic | 1.26 (0.54-2.96) | 1.41 (0.76-2.62) | 1.68 (0.94-3.01) |  |
| Gabon | 0.35 (0.07-1.77) | 1.36 (0.55-3.39) | 0.65 (0.27-1.55) |  |
| Sao Tome & Principe | (omitted) | 0.18 (0.02-1.44) | 0.31 (0.04-2.61) |  |
| **South Africa** |  |  |  |  |
| Lesotho | 1.88 (0.50-6.98) | 0.94 (0.41-2.13) | 0.87 (0.34-2.22) |  |
| Namibia | 2.20 (0.59-8.17) | 1.24 (0.50-3.08) | 1.55 (0.73-3.33) |  |
| Swaziland | 1(omitted) | 1.77 (0.80-3.90) | 0.54 (0.21-1.39) |  |
| **South Asia** |  |  |  |  |
| Bangladesh | 1.78 (1.12-2.85)* | 1.74 (1.28-2.35)*** | 1.63 (1.24-2.14)*** |  |
| India | 2.18 (1.51-3.15)*** | 1.82 (1.46-2.26)*** | 1.45 (1.22-1.72)*** |  |
| Maldives | (omitted) | 25.04 (2.65-236.3)*** | 5.54 (1.89-16.25)*** |  |
| Nepal | 2.18 (0.83-5.7) | 2.00 (1.29-3.1)*** | 1.63 (1.09-2.45)* |  |
| Pakistan | 3.02 (1.48-6.16)*** | 1.74 (1.05-2.88) * | 1.34 (0.99-1.83) |  |
| **Southeast Asia** |  |  |  |  |
| Cambodia | 1.81 (0.31-10.39) | 1.88 (1.05-3.36)* | 1.54 (0.96-2.47) |  |
| Indonesia | 11.2 (3.69-33.81)*** | 2.04 (1.08-3.82* | 1.51 (0.80-2.87) |  |
| Timor-Leste | 2.70 (0.61-11.85) | 1.36 (0.42-4.42) | 1.83 (1.00-3.34) |  |
| **Caribbean and Latin America** | |  |  |  |
| Bolivia | 2.62 (0.98-6.98)* | 0.93 (0.44-1.95) | 1.60 (0.44-2.70) |  |
| Guyana | 8.73 (2.1-36.41)*** | 1.11 (0.23-5.43) | 0.75 (0.21-2.66) |  |
| Dominican Republic | 1.63 (0.62-4.26) | 1.01 (0.43-2.34) | 0.75 (0.40-1.42) |  |
| Haiti | 1.34 (0.32-5.61) | 1.34 (0.80-2.25) | 0.84 (0.48-1.46) |  |
| Honduras | 1.52 (0.8-2.89) | 1.43 (0.93-2.21) | 1.17 (0.78-1.76) |  |
| **Notes:** |  |  |  |  |
| Significance level: *p<0.05; **p<0.01; ***p<0.005 |  |  |  |  |
| O.R.: Odds Ratio | |  |  |  |
| C.I.: Confidence Intervals |  |  |  |  |
| R.C.: Reference category |  |  |  |  |
