# Supplementary material for: The impact of young maternal age at birth on neonatal mortality: Evidence from 45 low and middle income countries
Source: PLoS One. 2018 May 23;13(5):e0195731. doi: 10.1371/journal.pone.0195731 (PMC5965834; doi:10.1371/journal.pone.0195731)
Supplement: S3 Table — (DOCX) [file pone.0195731.s003.docx]

**Supplementary 3 Table**: Number of deaths and births by large regional grouping (unweighted).

|  | Sub-Saharan Africa | | Southeast Asia | | Latin America & Caribbean | |
| --- | --- | --- | --- | --- | --- | --- |
|  | Deaths | Total Births | Deaths | Total Births | Deaths | Total Births |
| <15 | 145 | 2,338 | 25 | 507 | 17 | 703 |
| 15 | 240 | 4,825 | 80 | 1,200 | 34 | 1,573 |
| 16 | 449 | 9,526 | 132 | 2,315 | 45 | 2,868 |
| 17 | 595 | 14,056 | 215 | 4,134 | 72 | 3,949 |
| 18 | 618 | 17,404 | 272 | 5,986 | 75 | 4,768 |
| 19 | 682 | 19,772 | 334 | 7,958 | 86 | 5,088 |
| 20-29 | 5,950 | 215,199 | 2,343 | 85,180 | 6,79 | 44,720 |
