# Supplementary material for: The impact of young maternal age at birth on neonatal mortality: Evidence from 45 low and middle income countries
Source: PLoS One. 2018 May 23;13(5):e0195731. doi: 10.1371/journal.pone.0195731 (PMC5965834; doi:10.1371/journal.pone.0195731)
Supplement: S4 Table — (DOCX) [file pone.0195731.s004.docx]

**Supplementary 4 Table:** Percentage distribution of background factors by large regional grouping (weighted)

|  |  | Sub-Saharan Africa | Southeast Asia | Latin America & Caribbean |
| --- | --- | --- | --- | --- |
| Place of residence | Urban | 27.7 | 26.0 | 56.7 |
|  | Rural | 72.4 | 74.1 | 43.3 |
| Birth order | First | 29.6 | 37.8 | 43.5 |
|  | 2-3 | 57.1 | 54.8 | 51.3 |
|  | 4+ | 13.3 | 7.4 | 5.2 |
| Birth Interval | <18 months | 6.2 | 8.6 | 6.7 |
|  | >18 months | 93.8 | 91.4 | 93.3 |
| Education | None | 46.5 | 36.5 | 5.3 |
|  | Primary | 32.6 | 23.9 | 43.2 |
|  | Secondary+ | 20.9 | 39.6 | 51.5 |
| Place of delivery | Home | 46.2 | 60.2 | 21.3 |
|  | Facility | 53.8 | 39.8 | 78.7 |
| Antenatal care | None | 9.6 | 13.3 | 3.8 |
|  | 1-3 visits | 20.2 | 24.1 | 8.2 |
|  | 4 or more | 31.1 | 32.0 | 62.8 |
|  | Missing | 39.1 | 30.6 | 25.2 |
| Count |  | 283,120 | 107,280 | 63,669 |
